# Genetic Characterization of a Linezolid- and Penicillin-Resistant *Enterococcus hirae* Isolate Co-Harboring *poxtA* and *pbp5fm*

**DOI:** 10.1155/tbed/9275403

**Published:** 2025-07-13

**Authors:** Jinhu Huang, Aijuan Li, Mengli Wang, Shushu Gu, He Hu, Xiaoming Wang, Liping Wang

**Affiliations:** ^1^MOE Joint International Research Laboratory of Animal Health and Food Safety, Risk Assessment Center of Veterinary Drug Residue and Antimicrobial Resistance, Center for Veterinary Drug Research and Evaluation, College of Veterinary Medicine, Nanjing Agricultural University, Nanjing 210095, China; ^2^Nanjing Animal Husbandry and Veterinary Station, Nanjing 210012, China

**Keywords:** *E. hirae*, IS*1216*, MDR, *pbp5fm*, *poxtA*

## Abstract

Linezolid and penicillin are critical for treating multidrug resistant (MDR) Gram-positive infections, but the emergence of resistance to both seriously threatens public health. Here, we first report the cocarrying *poxtA* (oxazolidinone resistance) and *pbp5fm* (β-lactam resistance) genes by the plasmid in a strain of *Enterococcus hirae* HDC14-2 derived from porcine. The isolate also exhibits MDR phenotypes to phenicols, oxazolidinones, tetracyclines, β-lactams, aminoglycosides, macrolides, and lincosamides. Whole-genome sequencing (WGS) revealed these resistance genes, along with *tet*(*L*), *tet*(*M*), *catA*, *erm*(B), *aac*(*6*)-*aph*(*2”*), *aadE*, *spw*, *lsa*(E), *lnu*(B), *sat4*, and *aphA3*, were clustered in a novel MDR region flanked by IS*1216* elements on plasmid pHDC14-2.133K. This IS*1216*-bounded MDR region formed translocatable units (TUs), including an IS*1216*-*poxtA* TU that was also identified on a secondary plasmid, pHDC14-2.27K. Functional assays demonstrated the excisability and mobility of these TUs, indicating its potential ability integration into other plasmids or chromosomes. Critically, electrotransformation confirmed the transfer of pHDC14-2.27K (*poxtA*-carrying) to *Enterococcus faecalis* JH2-2, with retained TU activity and minimal fitness cost. This study provides the evidence of colocalized *poxtA* and *pbp5fm* on plasmids in enterococci, highlighting their role in disseminating pan-resistance among bacteria. Although *E. hirae* is not an important pathogenic bacterium to humans and animals, but its potential risk to horizontally spread of these resistance genes important in medicine still cannot be ignored.

## 1. Introduction

Though enterococci usually emerged as opportunistic pathogens associated with hospital-acquired infections, recently, an increasing outbreak of *Enterococcus hirae*-associated endocarditis affecting young broiler breeders is presented, which causes economic losses to the poultry industry [1]. The increasing use of antibiotics in veterinary settings has contributed to the emergence of MDR *E. hirae*, posing a significant threat to public health [2, 3]. As is well known, enterococci usually resistant to numerous antibiotics, including β-lactams and vancomycin. Resistance to penicillins in enterococci is often acquired through horizontal gene transfer, involving low-affinity penicillin-binding proteins (PBPs) or mutations in PBP-encoding genes [4–6]. Notably, PBP5-mediated penicillin resistance has become a global issue in nosocomial infections, with over 90% of clinical isolates showing ampicillin resistance [4]. Moreover, the cotransfer of *pbp5* with vancomycin-resistant genes (e.g., *vanB*) drives the spread of multidrug-resistant (MDR) strains [7]. Studies reveal that *Enterococcus faecium* isolates with high-level ampicillin resistance are related with overexpress of low-affinity PBP5, encoded by *pbp5fm*, reducing penicillin susceptibility [8–10]. The *pbp5fm* gene is prevalent in clinical *E. faecium* strains and has been recently detected in some *E. faecalis* and *E. hirae* isolates [11–15].

Linezolid, an oxazolidinone antibiotic, is a last resort treatment for infections caused by clinically significant Gram-positive pathogens, such as vancomycin-resistant enterococci (VRE) and methicillin-resistant *Staphylococcus aureus* (MRSA) [16]. However, the increasing prevalence of linezolid resistance poses a significant clinical and public health challenge [17, 18]. Linezolid resistance typically arises from mutations in domain V of the 23S rRNA (e.g., G2576U) or the acquisition of horizontally transferred genes, such as *cfr* (and its variants *cfr* [B/C/D/E]) and *optrA* [19, 20]. These genes drive cross-resistance to multiple antibiotic classes: *cfr* confers resistance to PhLOPSA agents (phenicols, lincosamides, oxazolidinones, pleuromutilins, and streptogramin A) [21–24], while *optrA* specifically mediates resistance to oxazolidinones and phenicols [25–28]. Recently, another oxazolidinone resistance gene, *poxtA*, was identified. This gene encodes an ABC-F protein distantly related to OptrA and confers elevated minimum inhibitory concentrations (MICs) to oxazolidinones, phenicols, and tetracyclines. PoxtA has been primarily identified in animal-associated bacteria, including MRSA and enterococci, under selective pressure [29, 30]. Notably, linezolid resistance exhibits cross-species distribution with multiple co-occurring resistance genes, including *optrA*, *cfr*, *cfr* (D), and *poxtA2* in resistant enterococci and *optrA-fexA* coexistence in Campylobacter [31, 32].

The coexistence of *poxtA* and *pbp5fm* in the same strain has not been reported, though they were being identified separately in previous studies. In this study, we investigated an *E. hirae* isolate resistant to both linezolid and penicillin medicated by *poxtA* and *pbp5fm* genes colocating on mobile genetic elements (MGEs), hinting the potential risks of horizontal spread of resistance genes important in medicine. The presence of MDR *E. hirae* in food-producing animals underscores the need for continued surveillance and control measures to mitigate the spread of antimicrobial resistance (AMR).

## 2. Materials and Methods

### 2.1. Bacterial Isolation and Antimicrobial Susceptibility Testing

*Enterococcus hirae* HDC14-2 was isolated from swine fecal samples from a farm in Jiangsu, China, in 2020. Strains were grown in Tryptic soy broth (TSB) or agar (TSA) plates at 37 °C unless otherwise stated. Antimicrobial susceptibility testing was performed by the broth microdilution method, and results were interpreted according to the Clinical and Laboratory Standards Institute standard document M100-ED33 [33]. *E. faecalis* ATCC 29212 served as the quality control strain. The presence of oxazolidinone resistance genes *cfr*, *cfr* (B), *cfr* (C), *optrA*, and *poxtA* were screened by PCR using the primers listed in Table 1.

### 2.2. Whole-Genome Sequencing and Analysis

The bacterial genomic DNA was purified and subjected to whole-genome sequencing (WGS,) using the Illumina HiSeq2000 platform plus PacBio RS II sequencing technology (Shanghai Biozeron Co. Ltd). The complete sequences of chromosomes and plasmids were annotated using the NCBI Prokaryotic Genome Annotation Pipeline. Acquired resistance genes were identified by ResFinder 3.1 (https://cge.cbs.dtu.dk/services/ResFinder/). Genetic comparison of the resistance genes was analyzed using the BLAST programs (http://blast.ncbi.nlm.nih.gov/Blast.cgi) and visualized using EasyFig 2.2.3 [34].

### 2.3. Transfer Experiments, S1-PFGE and Hybridization

Conjugation was performed by filter-mating assays as previously described by using *E. faecalis* JH2-2 as a recipient [35]. Selections of transcongugants were plated on TSA plates containing linezolid (2 mg/L), rifampicin (50 mg/L), and fusidic acid (50 mg/L). For the strain that failed to transfer in the conjugation experiment, the plasmids were extracted, and then transferred by electrotransformation (2.5 kV, 200 X, 25 μF). *E. hirae* HDC14-2, *E. faecalis* JH2-2, and the corresponding electrotransformation were digested with SmaI, and a *Salmonella* strain H9812 digested by XbaI was used for molecular weight and size standard. For Southern blot hybridization, the DNA probes were amplified from the genome of isolate AH0906 by primer pairs (Table 1) targeting the *poxtA* gene and labeled with nonradioactive digoxigenin (DIG) using DIG High Prime DNA Labeling and Detection Starter Kit I (Roche Diagnostics GmbH, Germany) according to the kit instructions. Subsequent hybridization and visualization were performed using the above kit.

### 2.4. IS*1216*-Mediated Excision Assays

The presence of the circular intermediates probably resulted from IS*1216*-mediated excision, also known as translocatable units (TUs), and was detected by using specifically designed primers P1-P12 (Table 1), followed by Sanger sequencing.

### 2.5. Fitness Assays

The fitness was calculated by comparing the differences of recipient and transformant in vitro growth and competition assays. In vitro growth assays, strains were grown in TSB overnight, diluted to an OD_600_ of 0.02, and OD_600_ values were tested every hour in TSB for successive 24 h at 37°C with shaking at 180 rpm. In vitro competition assays, competitors were mixed in 1:1 ratio and diluted 1:100 in 5 mL daily for 7 days. The CFU of the competitors at both the startpoint (0 h) and the endpoint (24 h) was counted by plating onto medium with or without linezolid. The relative fitness (*w*) was determined in competition experiments in 10 replicate and repeated at least twice, as previously described [36].

### 2.6. Plasmid Stability Assays

The plasmid stability assays were performed as described previously with minor modifications [37]. Briefly, a single clone of the transformant was grown in TSB without antibiotics. The cultures were then serially cultivated every day with 1:1000 dilution in antibiotic-free TSB for 30 days with 10 duplicates. A total of 100 colonies were randomly selected from the plates and then cultured with or without antibiotics. Plasmid stability was evaluated by dividing the number of colonies growing on the antibiotic broth by the total number of colonies growing on the antibiotic-free broth.

### 2.7. Nucleotide Sequence Accession Number

The complete sequences of *E. hirae* HDC14-2 and its plasmids have been deposited to GenBank under Accession Numbers CP042289-CP042294.

## 3. Results

### 3.1. Identification of a Linezolid- and Penicillin-Resistant *Enterococcus hirae* Isolate

**Figure 1:**
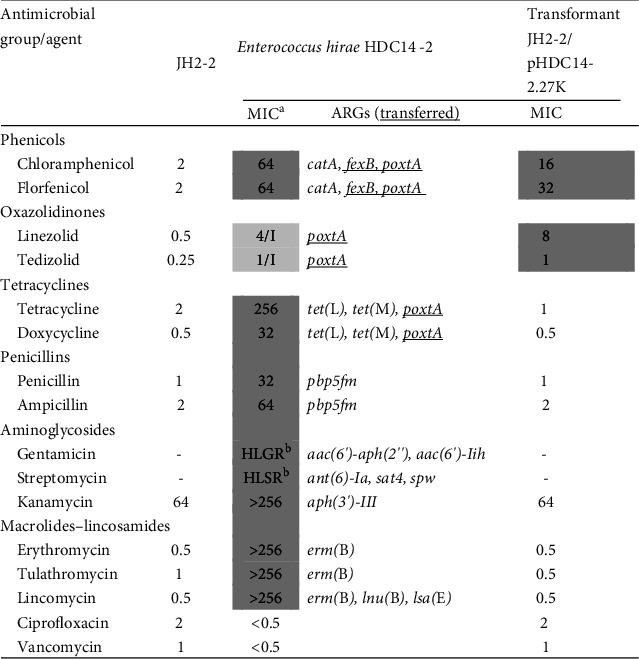
Characterization of *E. hirae* HDC14-2 and transformant JH2-2/pHDC14-2.27K. ^a^The MICs (mg/L) in gray shadow represent that strains were nonsusceptible to corresponding antimicrobial agents. ^b^HLGR, high-level gentamicin resistance; HLSR, high-level streptomycin resistance. “-”, not for HLGR/HLSR. Underlining indicates that antibiotic resistance genes were transferable.

A linezolid- and penicillin-resistant *Enterococcus* isolate, designated HDC14-2, was identified from swine fecal samples (0.45% prevalence) collected in Jiangsu, China, in 2020 as part of the National Antimicrobial Resistance Monitoring and Surveillance Program in Animals of China. 16S rRNA sequencing analysis confirmed its classification as *E. hirae*. Antimicrobial susceptibility testing revealed that HDC14-2 exhibited resistance to multiple antibiotic classes, including phenicols (chloramphenicol and florfenicol), oxazolidinones (linezolid and tedizolid), tetracyclines (tetracycline and doxycycline), penicillins (penicillin and ampicillin), aminoglycosides (gentamicin, streptomycin, and kanamycin), macrolides (erythromycin and tulathromycin), lincomycin, while remaining susceptible to ciprofloxacin and vancomycin (Figure 1). PCR detection and WGS analysis identified the coexistence of *poxtA* and *pbp5fm* genes, along with 13 additional resistance determinants, including the phenicols resistance genes *fexB* and *catA*, the tetracyclines resistance genes *tet* (L) and *tet* (M), the macrolides-lincosamides-streptogramin B resistance genes *erm* (B), *lnu* (B), and *lsa* (E), and the aminoglycosides resistance genes *aac*(*6′*)-*Iih*, *aac*(*6′*)-*aph*(*2′′*), *spw*, *aadE*, *sat4*, and *aphA3* (Figure 1). The AMR genotype was consistent with the observed MICs.

### 3.2. Characterization of *poxtA*- and *pbp5fm*-Carrying Plasmid in *Enterococcus hirae* HDC14-2

**Figure 2:**
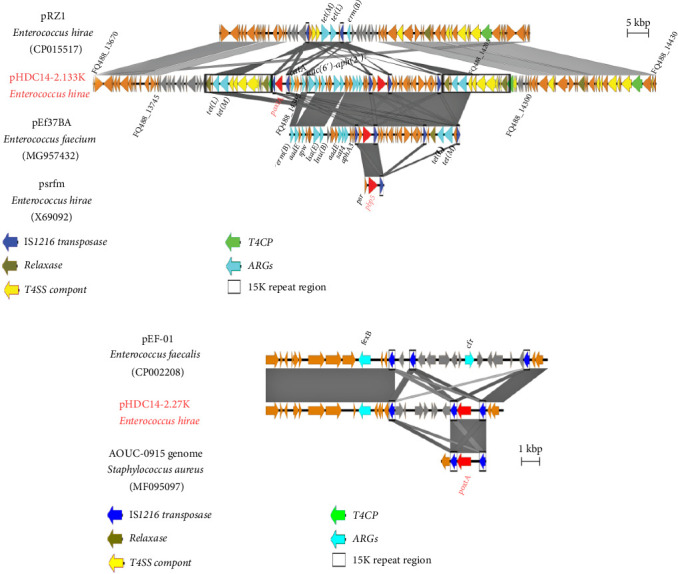
Characterization of *poxtA* and *pbp5fm* carrying plasmids in *E. hirae* HDC14-2. (A) Linear schematic diagram of pHDC14-2.133K in this study and prz1 in *E. hirae*. And comparison of the genetic contexts of *pbp5fm* in plasmids pHDC14-2.133K with corresponding sequences in the plasmids of *E. faecium* (pEf37BA) and *E. hirae* (psrfm). (B) Linear schematic diagram of pHDC14-2.27K in this study and pEF-01 in *E. faecium*. comparison of the genetic contexts of *poxtA* in plasmids pHDC14-2.77K with corresponding sequences in the genomes of *S. aureus* AOUC-0915. The position and transcription direction of the ORF are indicated by arrows. The gray shadow represents homologous regions, and the darker the color, the higher the nucleotide homology. Transposase, relaxse, T4SS compont, T4CP, and ARGs are represented in deep blue, brown, yellow, green, and light blue, respectively. Orange arrows indicate identical genes, gray arrows indicate different genes, and red arrows indicate *pbp5* and *poxtA* resistance genes.

Complete genome sequencing of the *E. hirae* HDC14-2 revealed the presence of five plasmids with sizes ranging from 27,303- to 133,362-bp (Table 2). The resistance genes were predominantly located on two plasmids, pHDC14-2.133K and pHDC14-2.27K, except for *aac*(*6′*)-*Iih*, which was found in the chromosomal genome (Table 3). Plasmid pHDC14-2.133K harbored a mosaic structure (Figure 2A), encoding multiple resistance genes including *poxtA*, *pbp5fm*, along with *tet* (*L*), *tet* (*M*), *catA*, *aac* (*6'*)-*aph* (*2”*), *erm* (B), *aadE*, *spw*, *lsa* (E), *lnu* (B), *sat4*, and *aphA3*. Comparative analysis of plasmid pHDC14-2.133K revealed its chimeric nature. The plasmid appears to have originated from a recombination event involving the backbone of the *E. hirae* plasmid pRZ1 (GenBank: CP015517) and the multidrug resistance (MDR) region of the *E. faecium* plasmid pEf37BA (GenBank: MG957432). The pHDC14-2.133K backbone region (from FQ488_14300 to FQ488_14430 and FQ488_13670 to FQ488_13745, ~45.46 kb) shared 91.75% sequence identity with pRZ1, while the MDR region exhibited significant structural divergence (Figure 2A). This MDR region, flanked by two identical 15K repeat regions containing *tet* (*L*), *tet* (*M*), relaxase, and type IV secretion system (T4SS) components, carried a transposon containing *poxtA* and two IS*1216* elements. Notably, the MDR region upstream of the IS*1216*-*poxtA*-carrying transposon exhibited a high degree of similarity to the *E. faecium* plasmid pEf37BA, suggesting extensive horizontal gene transfer between these species. Additionally, the *pbp5fm* gene was found to be flanked by two IS*1216* copies in the same orientation within the MDR region (Figure 2A). The IS*1216*-*pbp5fm*-carrying transposon in plasmid pHDC14-2.133K showed a high degree of identity with parts of the *E. faecium* plasmid pEf37BA and *E. hirae* plasmid psrfm, but differed in that *pbp5fm* was flanked only by a single copy of IS*1216* in *E. hirae* psrfm (Figure 2A).

Plasmid pHDC14-2.27K, a smaller plasmid, also carried an IS*1216*-*poxtA* transposon and with 58% homology to *E. faecalis* plasmid pEE-01(GenBank: CP002208), except for the presence of *poxtA* in pHDC14-2.27K instead of the *cfr* gene. And the IS*1216*-*pbp5fm*-carrying transposon in plasmid pHDC14-2.27K showed a high degree of identity with parts of the *S. aureus* AOUC-0915 genome (Figure 2B).

### 3.3. Transfer of the Novel poxtA-Carrying Plasmids Between Enterococci

**Figure 3:**
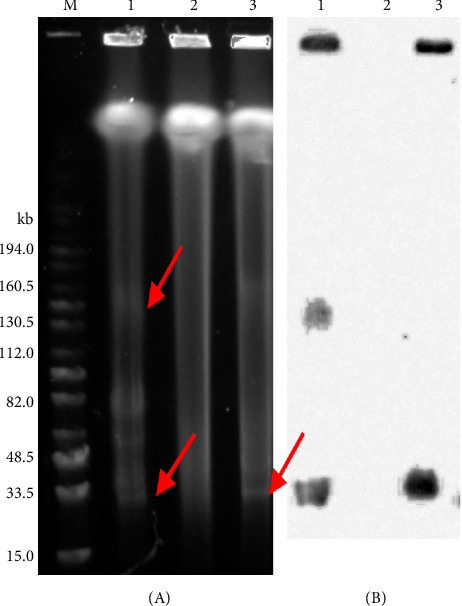
S1-PFGE (A) and Southern blot hybridization with *poxtA*-specific probe (B) of the donor *E. hirae* HDC14-2, the recipient *E. faecalis* JH2-2, and the transformant *E. faecalis* JH2-2/pHDC14-2.27K. Locations of the *poxtA*-carrying plasmids are highlighted by red arrows in the (A). Lane 1, *E. hirae* HDC14-2; lane 2, *E. faecalis* JH2-2; lane 3, *E. faecalis* JH2-2/pHDC14-2.27K.

The potential for horizontal gene transfer of resistance determinants was assessed using *E. hirae* HDC14-2 as the donor and *E. faecalis* JH2-2 as the recipient strains. Conjugation assays failed to transfer linezolid and ampicillin resistance; however, electrotransformation successfully introduced the *poxtA*-carrying plasmid pHDC14-2.27K into *E. faecalis* JH2-2. The presence of the *poxtA* gene, but not *pbp5fm* was confirmed in transformants. S1-PFGE and Southern hybridization demonstrated that *poxtA* was transferred as part of the entire pHDC14-2.27K plasmid rather than as a TUs (Figure 3).

MIC testing of the transformants revealed resistance to chloramphenicol, florfenicol, linezolid, tedizolid, and kanamycin, but susceptibility to penicillins and tetracyclines. The MIC for phenicols and oxazolidinones was higher in the transformed strain than in the recipient strain. Interestingly, the introduction of *poxtA* into the recipient bacteria did not increase the MIC of tetracyclines, consistent with previous studies [38] (Figure 1).

### 3.4. IS*1216* Elements Mediate the Generation of *poxtA* and *pbp5fm* Carrying TUs

**Figure 4:**
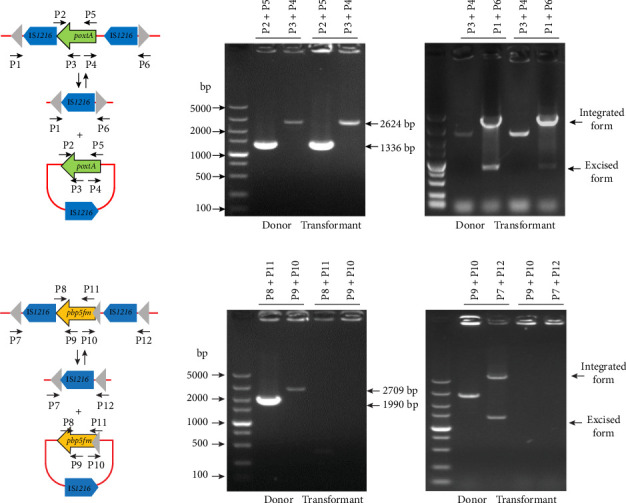
Activity detection of IS*1216* TUs carrying the resistance genes *poxtA* and *pbp5fm*. (A) Excised form and the circular intermediates of IS*1216*-*poxtA* TU. (B) Excised form and the circular intermediates of IS*1216*-*pbp5fm* TU.

A total of eight IS*1216* copies were detected within the two plasmids carrying *poxtA* and *pbp5fm*, with flanking IS*1216* elements in the same orientation around each gene. Reverse PCR assays confirmed the excision of two TUs: TU1 (IS*1216*-*poxtA*) and TU2 (IS*1216*-*pbp5fm*), indicating that these elements can circularize and potentially integrate into other genomic locations (Figure 4A,B). Similar activity was observed in transformants, demonstrating that IS*1216*-mediated recombination events facilitate the mobility of resistance genes among Gram-positive bacteria.

### 3.5. Fitness Cost of Plasmid pHDC14-2.27K

**Figure 5:**
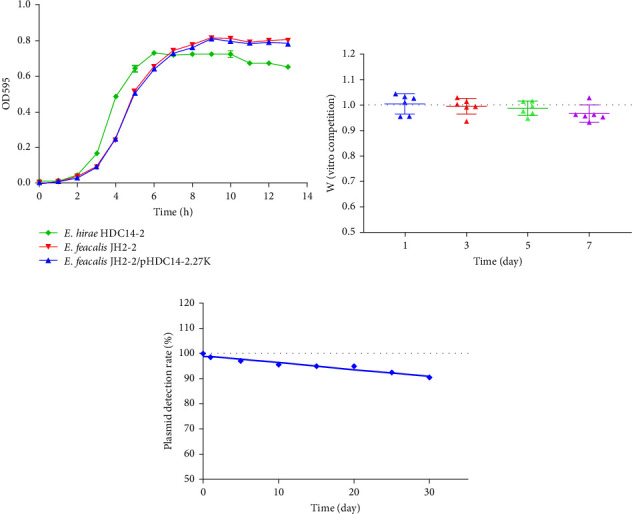
Fitness cost of plasmid pHDC14-2.27K. (A) Growth curves of *E. hirae* HDC14-2, transformant JH2-2/pHDC14-2.27K and *E. faecalis* JH2-2 under the same conditions in vitro. (B) Relative competitive fitness (W) of transformant versus recipient strain during 7-day coculture in antibiotic-free TSB. Dashed line indicates fitness parity (*W* = 1). (C) Plasmid retention rate during 30-day serial passaging without antibiotic selection.

To evaluate the potential fitness burden imposed by the acquisition of pHDC14-2.27K, growth curve analyses were conducted. Results indicated no significant impact on the growth rate of *E. faecalis* JH2-2 transformants compared to the wild type strain (Figure 5A). In vitro competition assays, however, revealed a competitive disadvantage for the transformant compared to the recipient strain, and the competitive index decreased by an average of 3.7% over the duration of 7 days of coculture (Figure 5B). This suggests that the presence of pHDC14-2.27K may impose a metabolic burden, limiting the bacterium's ability to compete for resources in the absence of antibiotic selection pressure. Plasmid stability assays showed that after 30 days of serial passaging without selective pressure, the plasmid retention rate was 89% after 30 days (Figure 5C), indicating a gradual loss of the plasmid over time in the absence of antibiotic selection.

## 4. Discussion

The identification of *E. hirae* HDC14-2, harboring both *poxtA* and *pbp5fm* resistance genes, underscores the growing threat of MDR enterococci in zoonotic reservoirs. This dual resistance mechanism—conferring resistance to oxazolidinones (via *poxtA*) and β-lactams (via *pbp5fm*)—represents a novel challenge in antimicrobial stewardship. Notably, this study provides the first evidence of their coexistence in a single *E. hirae* isolate, amplifying concerns about synergistic resistance dissemination in clinical settings.

The *pbp5fm* gene, embedded within an IS*1216*-flanked transposon on plasmid pHDC14-2.133K, mirrors genetic architectures observed in *E. faecium* and *E. faecalis* [9, 39], suggesting a conserved interspecies transmission pathway. IS*1216*-mediated recombination likely facilitates the integration of *pbp5fm* into diverse MGEs, as evidenced by structural parallels with Tn*5382*/Tn*916* transposons historically linked to β-lactam resistance spread [9, 40–42]. Similarly, *poxtA* exhibits mobility through IS*1216*-driven transposition, forming mosaic plasmids capable of cross-genus transfer. Comparative analysis of IS*1216*-*poxtA* loci across taxa—including *S. aureus* AOUC-0915 (MF095097), *Lactobacillus* spp. BCC1 (CP018763), and *E. faecalis* P36 (KP834591)—reveals conserved transposon frameworks with orientation and copy number variations [29, 43]. In *S. aureus* AOUC-0915, the motifs are identical, and in particular, in *Lactobacillus* BCC1, the IS*1216* present upstream of the *poxtA* gene is orientated in the opposite direction, whereas in *E. faecalis* P36, only a single copy of IS*1216* is present downstream of the *poxtA* gene. These observations underscore the remarkable adaptability of these MGEs in establishing themselves within diverse genomic environments.

The colocalization of *poxtA* and *pbp5fm* on distinct MGEs within pHDC14-2.133K implies a modular gene transfer system. IS*1216* elements act as molecular “bridges,” enabling cyclization of TUs and subsequent integration into recipient genomes [35, 39]. Experimental evidence of TU excision from pHDC14-2.133K supports this model, though a slight fitness cost in transformants suggests a trade-off between resistance acquisition and ecological competitiveness. This fitness burden may paradoxically limit short-term transmission, while allowing long-term persistence of resistance reservoirs under antibiotic selection.

Although this study provides important insights into the coexistence of *poxtA* and *pbp5fm* on MGEs, some limitations must be recognized. The analysis was limited to single isolates, which may not fully represent the genetic diversity and transmission dynamics of these resistance determinants in natural bacterial populations. Despite these limitations, our findings have important implications for public health surveillance. The identification of these clinically relevant resistance genes on metastable genetic elements of enterococci highlights potential pathways for the spread of MDR.

## 5. Conclusions

In conclusion, this study reports the first cooccurrence of *poxtA* (oxazolidinone resistance) and *pbp5fm* (β-lactam resistance) in *E. hirae*, mediated by IS*1216*-flanked transposons on a mosaic plasmid. IS*1216*-driven mobilization of these genes via TUs highlights their potential for cross-genus dissemination. The detection of MDR *E. hirae* in livestock underscores the One Health ramifications of veterinary antibiotic overuse. Future research should prioritize ecological drivers of IS*1216* recombination and evolutionary trade-offs between resistance acquisition and bacterial fitness.

## Figures and Tables

**Table 1:** Primers used in this study.

	Primer	Sequence (5′ to 3′)
For mobile oxazolidinone resistance genes detection		

	cfr-F	TGAAGTATAAAGCAGGTTGGGAGTCA
	cfr-R	ACCATATAATTGACCACAAGCAGC
	cfrB-F	CCGCATCCGTGAACTAACAG
	cfrB-F	GCTGGTTGGGAGTCATTTTGT
	cfrC-F	GGTGAAACTGTTGTGGAGAT
	cfrC-R	AGTTTCCGTAACTGTCGTTT
	optrA-F	AGGTGGTCAGCGAACTAA
	optrA-R	ATCAACTGTTCCCATTCA

For detection of IS*1216*-mediated excision of translocatable unit (TU)		

P1	OUT-TU-poxtA-1	TCTTTACTTAACCCGTGGTG
P2	poxtA-F	TCCACAAAGGATGGGTTATG
P3	TU-poxtA-inv-F	AACCATTATTGCCGTTAGCC
P4	TU-poxtA-inv-R	ACAATCGCCATTTCACGAA
P5	poxtA-R	ATGCCCGTATTGGTTATCTC
P6	OUT-TU-poxtA-2	ATAAATATAACCAGCCGTGT
P7	OUT-TU-PBP5-1	GAAGTTTACTACCAGGTTGC
P8	PBP5-F	AAAATCGAACAGGCGCTT
P9	TU-PBP5-inv-F	GCAGCTTTGTTATAATCTCCT
P10	TU-PBP5-inv-R	GATGATTCAGCAACTAAACGA
P11	PBP5-R	TATTGTAATAGTTCGGGTGCT
P12	OUT-TU-PBP5-2	CTGATCAATATGCTGTAACGA

For *poxtA* DNA hybridization		

	poxtA-probe-F	GATTTCTCTTTTGCAGCTCT
	poxtA-probe-F	ATTCTATTGTTGGATGAGCCTA

**Table 2:** Features of the *Enterococcus hirae* isolate HDC14-2.

Replicon	Size (bp)	GC content (%)	CDs	Accession
Chromosome	2,895,174	36.89	2642	CP042289
pHDC14-2.133K	133,362	35.64	153	CP042290
pHDC14-2.74K	73,641	32.56	72	CP042291
pHDC14-2.69K	69,329	33.33	73	CP042292
pHDC14-2.35K	35,046	37.00	44	CP042293
pHDC14-2.27K	27,303	34.86	26	CP042294

**Table 3:** List of resistance genes identified in *Enterococcus hirae* isolate HDC14-2.

Replicon	Gene	Locus-tag	Location	Antibiotics to which resistance is known to be conferred
Chromosome	*aac* (*6′*)-*Iih*	FQ488_11125	2,348,704–2,349,252	Enzymic modification to gentamicin C1a, tobramycin, and netilmicin
pHDC14-2.133K	*tet* (L)	FQ488_13825	28,389–29,765	Tetracyclines
	*tet* (M)	FQ488_13835	29,959–31,878	Tetracyclines
	***poxtA***	FQ488_13905	43,129–44,757	Phenicols, oxazolidinones, tetracyclines^a^
	*catA*	FQ488_13920	47,040–47,687	Chloramphenicol
	*erm* (B)	FQ488_13945	49,593–50,330	Macrolides, lincosamides, streptogramin B
	*aac* (*6′*)-*aph* (*2*″)	FQ488_13965	51,157–52,596	Aminoglycosides, with the exception of streptomycin.
	*ant* (*6*)-*Ia* (*aadE*)	FQ488_13970	52,764–53,518	Streptomycin
	*spw*	FQ488_13980	54,221–55,030	Spectinomycin
	*lsa* (E)	FQ488_14000	57,097–58,581	Lincosamides, streptogramin A
	*lnu* (B)	FQ488_14005	58,635–59,438	Lincosamides
	*ant* (*6*)-*Ia* (*aadE*)	FQ488_14025	61,587–62,495	Streptomycin
	*sat4*	FQ488_14030	62,492–63,043	Streptothricin
	*aph* (*3′*)-*III*	FQ488_14035	63,127–63,921	Kanamycin
	***pbp5fm***	FQ488_14075	67,366–69,402	Penicillin, ampicillin
	*tet* (*L*)	FQ488_14180	84,946–86,322	Tetracyclines
	*tet* (*M*)	FQ488_14190	86,516–88,435	Tetracyclines
pHDC14-2.27K	*fexB*	FQ488_15420	10,637–12,046	Chloramphenicol and florfenicol
	***poxtA***	FQ488_15490	21,939–23,567	Phenicols, oxazolidinones, tetracyclines^a^

*Note*: The plasmid-borne oxazolidone resistance gene poxtA and β-lactam resistance gene pbp5fm were highlighted in bold.

^a^Resistance to tetracyclines was not observed in transformant *E. faecalis* JH2-2/pHDC14-2.27K that carrying *poxtA* gene.

## Data Availability

The genomic data supporting this study have been deposited in the NCBI database under Accession Number CP042289. These data are publicly available at https://www.ncbi.nlm.nih.gov/nuccore/CP042289.1

## References

[1] Manders T. T. M., Parmentier H. K., Papanikolaou A., Gallardo R. A. (2024). *Enterococcus hirae*-Associated Endocarditis Outbreak in Young Broiler Breeders of the Female Line. *Avian Diseases*.

[2] Sapunaric F., Franssen C., Stefanic P., Amoroso A., Dardenne O., Coyette J. (2003). Redefining the Role of *Psr* in Beta-Lactam Resistance and Cell Autolysis of *Enterococcus hirae*. *Journal of Bacteriology*.

[3] Mangan M. W., McNamara E. B., Smyth E. G., Storrs M. J. (1997). Molecular Genetic Analysis of High-Level Gentamicin Resistance in *Enterococcus hirae*. *Journal of Antimicrobial Chemotherapy*.

[4] Gagetti P., Bonofiglio L., Gabarrot G. G. (2019). Resistance to *β*-Lactams in Enterococci. *Revista Argentina de Microbiología*.

[5] Rice L. B., Carias L. L., Hutton-Thomas R., Sifaoui F., Gutmann L., Rudin S. D. (2001). Penicillin-Binding Protein 5 and Expression of Ampicillin Resistance in *Enterococcus faecium*. *Antimicrobial Agents and Chemotherapy*.

[6] Zorzi W., Zhou X. Y., Dardenne O. (1996). Structure of the Low-Affinity Penicillin-Binding Protein 5 PBP5fm in Wild-Type and Highly Penicillin-Resistant Strains of *Enterococcus faecium*. *Journal of Bacteriology*.

[7] Hanrahan J., Hoyen C., Rice L. B. (2000). Geographic Distribution of a Large Mobile Element That Transfers Ampicillin and Vancomycin Resistance Between *Enterococcus faecium* Strains. *Antimicrobial Agents and Chemotherapy*.

[8] Singh K. V., Galloway-Peña J., Montealegre M. C., Dong X., Murray B. E. (2024). Genomic Context as Well as Sequence of Both Psr and Penicillin-Binding Protein 5 Contributes to *β*-Lactam Resistance in *Enterococcus faecium*. *mBio*.

[9] Morroni G., Brenciani A., Litta-Mulondo A. (2019). Characterization of a New Transferable MDR Plasmid Carrying the *pbp5* Gene From a Clade B Commensal *Enterococcus faecium*. *Journal of Antimicrobial Chemotherapy*.

[10] Belhaj M., Boubaker I. B.-B., Redjeb S. B., Bouchami O. (2008). Molecular Characterisation of High-Level Ampicillin-Resistant *Enterococcus faecium* Isolates From Hospitalised Patients in Tunis. *International Journal of Antimicrobial Agents*.

[11] Montealegre M. C., Roh J. H., Rae M. (2017). Differential Penicillin-Binding Protein 5 (PBP5) Levels in the *Enterococcus faecium* Clades With Different Levels of Ampicillin Resistance. *Antimicrobial Agents and Chemotherapy*.

[12] Novais C., Tedim A. P., Lanza V. F. (2016). Co-Diversification of *Enterococcus faecium* Core Genomes and PBP5: Evidences of pbp5 Horizontal Transfer. *Frontiers in microbiology*.

[13] Gawryszewska I., Hryniewicz W., Sadowy E. (2012). Penicillin Resistance in *Enterococcus faecalis*: Molecular Determinants and Epidemiology. *Polish Journal of Microbiology*.

[14] Arbeloa A., Segal H., Hugonnet J. E. (2004). Role of Class A Penicillin-Binding Proteins in PBP5-Mediated Beta-Lactam Resistance in *Enterococcus faecalis*. *Journal of Bacteriology*.

[15] Ligozzi M., Pittaluga F., Fontana R. (1993). Identification of a Genetic Element (*psr*) which Negatively Controls Expression of *Enterococcus hirae* Penicillin-Binding Protein 5. *Journal of Bacteriology*.

[16] Mendes R. E., Deshpande L. M., Jones R. N. (2014). Linezolid Update: Stable in Vitro Activity Following More Than a Decade of Clinical use and Summary of Associated Resistance Mechanisms. *Drug Resistance Updates*.

[17] Deshpande L. M., Castanheira M., Flamm R. K., Mendes R. E. (2018). Evolving Oxazolidinone Resistance Mechanisms in a Worldwide Collection of Enterococcal Clinical Isolates: Results From the SENTRY Antimicrobial Surveillance Program. *Journal of Antimicrobial Chemotherapy*.

[18] Bender J. K., Cattoir V., Hegstad K. (2018). Update on Prevalence and Mechanisms of Resistance to Linezolid, Tigecycline and Daptomycin in Enterococci in Europe: Towards a Common Nomenclature. *Drug Resistance Updates*.

[19] Chen L., Han D., Tang Z., Hao J., Xiong W., Zeng Z. (2020). Co-Existence of the Oxazolidinone Resistance Genes *cfr* and *optrA* on Two Transferable Multi-Resistance Plasmids in One *Enterococcus faecalis* Isolate From Swine. *International Journal of Antimicrobial Agents*.

[20] Moure Z., Lara N., Marín M. (2020). Interregional Spread in Spain of Linezolid-Resistant *Enterococcus* spp. Isolates Carrying the *optrA* and *poxtA* Genes. *International Journal of Antimicrobial Agents*.

[21] Long K. S., Poehlsgaard J., Kehrenberg C., Schwarz S., Vester B. (2006). The *Cfr* rRNA Methyltransferase Confers Resistance to Phenicols, Lincosamides, Oxazolidinones, Pleuromutilins, and Streptogramin A Antibiotics. *Antimicrobial Agents and Chemotherapy*.

[22] Shen J., Wang Y., Schwarz S. (2013). Presence and Dissemination of the Multiresistance Gene *cfr* in Gram-Positive and Gram-Negative Bacteria. *Journal of Antimicrobial Chemotherapy*.

[23] Deshpande L. M., Ashcraft D. S., Kahn H. P. (2015). Detection of a New *cfr*-Like Gene, *cfr*(*B*), *Enterococcus faecium* Isolates Recovered From Human Specimens in the United States as Part of the SENTRY Antimicrobial Surveillance Program. *Antimicrobial Agents and Chemotherapy*.

[24] Hu Y., Won D., Nguyen L. P. (2022). Prevalence and Genetic Analysis of Resistance Mechanisms of Linezolid-Nonsusceptible Enterococci in a Tertiary Care Hospital Examined via Whole-Genome Sequencing. *Antibiotics (Basel)*.

[25] Wang Q., Peng K., Liu Z. (2023). Genomic Insights Into Linezolid-Resistant Enterococci Revealed Its Evolutionary Diversity and *poxtA* Copy Number Heterogeneity. *International Journal of Antimicrobial Agents*.

[26] Wang Y., Lv Y., Cai J. (2015). A Novel Gene, *optrA*, That Confers Transferable Resistance to Oxazolidinones and Phenicols and Its Presence in *Enterococcus faecalis* and *Enterococcus faecium* of Human and Animal Origin. *Journal of Antimicrobial Chemotherapy*.

[27] Li D., Wang Y., Schwarz S. (2016). Co-Location of the Oxazolidinone Resistance Genes *optrA* and *cfr* on a Multiresistance Plasmid From *Staphylococcus sciuri*. *Journal of Antimicrobial Chemotherapy*.

[28] Huang J., Chen L., Wu Z., Wang L. (2017). Retrospective Analysis of Genome Sequences Revealed the Wide Dissemination of *optrA* in Gram-Positive Bacteria. *Journal of Antimicrobial Chemotherapy*.

[29] Antonelli A., D’Andrea M. M., Brenciani A. (2018). Characterization of *poxtA*, a Novel Phenicol-Oxazolidinone-Tetracycline Resistance Gene From an MRSA of Clinical Origin. *Journal of Antimicrobial Chemotherapy*.

[30] Brenciani A., Fioriti S., Morroni G. (2019). Detection in Italy of a Porcine *Enterococcus faecium* Isolate Carrying the Novel Phenicol-Oxazolidinone-Tetracycline Resistance Gene *poxtA*. *Journal of Antimicrobial Chemotherapy*.

[31] Tang B., Wang Y., Luo Y. (2021). Coexistence of *optrA* and *fexA* in Campylobacter. *mSphere*.

[32] Tang B., Zou C., Schwarz S. (2023). Linezolid-Resistant *Enterococcus faecalis* of Chicken Origin Harbored Chromosome-Borne *optrA* and Plasmid-Borne *cfr*, *cfr*(D), and *poxtA2*, Genes. *Microbiology Spectrum*.

[33] CLSI (2023). *Performance Standards for Antimicrobial Susceptibility Testing*.

[34] Sullivan M. J., Petty N. K., Beatson S. A. (2011). Easyfig: A Genome Comparison Visualizer. *Bioinformatics*.

[35] Huang J., Wang M., Gao Y., Chen L., Wang L. (2019). Emergence of Plasmid-Mediated Oxazolidinone Resistance Gene *poxtA* From CC17 *Enterococcus faecium* of Pig Origin. *Journal of Antimicrobial Chemotherapy*.

[36] Starikova I., Al-Haroni M., Werner G. (2013). Fitness Costs of Various Mobile Genetic Elements in *Enterococcus faecium* and *Enterococcus faecalis*. *Journal of Antimicrobial Chemotherapy*.

[37] Nang S. C., Morris F. C., McDonald M. J. (2018). Fitness Cost of *mcr*-1-Mediated Polymyxin Resistance in *Klebsiella pneumoniae*. *Journal of Antimicrobial Chemotherapy*.

[38] Zhu Y., Yang W., Schwarz S. (2022). Characterization of an MDR *Lactobacillus salivarius* Isolate Harbouring the Phenicol-Oxazolidinone-Tetracycline Resistance Gene *poxtA*. *Journal of Antimicrobial Chemotherapy*.

[39] Raze D., Dardenne O., Hallut S., Martinez-Bueno M., Coyette J., Ghuysen J. M. (1998). The Gene Encoding the Low-Affinity Penicillin-Binding Protein 3r in, *Enterococcus hirae*, S185R Is Borne on a Plasmid Carrying Other Antibiotic Resistance Determinants. *Antimicrobial Agents and Chemotherapy*.

[40] Carias L. L., Rudin S. D., Donskey C. J., Rice L. B. (1998). Genetic Linkage and Cotransfer of a Novel, *vanB*-Containing Transposon (Tn*5382*) and a Low-Affinity Penicillin-Binding Protein 5 Gene in a Clinical Vancomycin-Resistant *Enterococcus faecium* Isolate. *Journal of Bacteriology*.

[41] Rice L. B., Carias L. L., Rudin S., Lakticová V., Wood A., Hutton-Thomas R. (2005). *Enterococcus faecium* Low-Affinity pbp5 Is a Transferable Determinant. *Antimicrobial Agents and Chemotherapy*.

[42] García-Solache M., Lebreton F., McLaughlin R. E., Whiteaker J. D., Gilmore M. S., Rice L. B. (2016). Homologous Recombination Within Large Chromosomal Regions Facilitates Acquisition of *β*-Lactam and Vancomycin Resistance in *Enterococcus faecium*. *Antimicrobial Agents and Chemotherapy*.

[43] Chen L., Hu J. X., Liu C. (2021). Identification of the Multiresistance Gene *poxtA* in Oxazolidinone-Susceptible *Staphylococcus haemolyticus* and *Staphylococcus saprophyticus* of Pig and Feed Origins. *Pathogens*.

